# Analysis of the integration of medical and nursing care and its association with depression and anxiety among medical staff in the Jinhua area

**DOI:** 10.3389/fpsyt.2025.1616682

**Published:** 2025-09-19

**Authors:** An Sheng, TaoTao Yu, YanLi Yu, MengDi Zhang, Ye Zeng, Ling Ye

**Affiliations:** Department of Geriatirics, Fifth Hospital of Jinhua City, Jinhua, Zhejiang, China

**Keywords:** Jinhua area, medical institutions, integration of medical and nursing care, depression, anxiety, medical staff

## Abstract

**Objective:**

To evaluate the relationship between implementation of the medical–nursing care integration model and the psychological health (depression and anxiety) of medical staff in the Jinhua area.

**Methods:**

A total of 1,022 elderly patients and 182 medical staff from four institutions in Jinhua were surveyed. Patient information, service utilization, and medical costs were collected, while staff familiarity with the Patient-Driven Payment Model (PDPM) and their mental health were assessed using the PHQ-9, GAD-7, and SCL-90 scales. Statistical analyses were conducted to explore associations between PDPM familiarity and psychological outcomes.

**Results:**

Most medical staff reported high familiarity with PDPM and successful adaptation to the integrated model. Despite this, nearly 10% of staff experienced symptoms of depression and 8% reported anxiety. Statistical analysis indicated that higher PDPM familiarity was significantly associated with lower rates of depression, anxiety, and overall psychological distress.

**Conclusion:**

The integration of medical and nursing care in Jinhua appears to improve not only the efficiency of elderly services but also supports the mental health of medical staff. However, psychological risks remain, highlighting the need for integrated care models to embed ongoing mental health support mechanisms for healthcare professionals.

## 1 Introduction

With the implementation of family planning policy and advances in medical technology, China has become the country with the largest elderly population and the fastest rate of aging (1). This trend poses stringent demands on the healthcare system, particularly with the rising need for chronic disease management, long-term care, and mental health services for older adults (2). The traditional Chinese model of elderly care has become insufficient to meet these complex needs (3), making the optimization of resource allocation through policy innovation and service integration a pressing priority.

Against this backdrop, the integrated medical and elderly care model has been promoted as a key strategy to improve service quality and reduce the social burden of aging (4). By consolidating medical and caregiving resources, this model seeks to provide comprehensive, continuous support for the elderly. However, its effective implementation requires adaptive reforms in service provision, staff training, and payment mechanisms (5).

The Patient-Driven Payment Model (PDPM), introduced as a new healthcare payment mechanism, has reshaped institutional practices by optimizing resource allocation and adjusting incentive structures. Studies have shown that its adoption influences staffing patterns and service intensity, aligning care more closely with patient needs (6). While PDPM was designed to achieve budget neutrality, its long-term effects on institutional efficiency and quality of care remain under evaluation (7).

In China, PDPM has been closely linked with the policy push toward integrated medical and elderly care. Its classification-based reimbursement enhances the precision of patient assessments and strengthens the capacity of medical teams through systematic training (8). At the same time, however, these reforms place new demands on healthcare workers. Increased training requirements, performance monitoring, and role adaptations may elevate occupational stress and affect the psychological well-being of medical staff (9). Since staff are central to delivering integrated care, their mental health directly influences both care quality and sustainability of the model.

Depression and anxiety were chosen as key variables because they are among the most prevalent mental health concerns in healthcare workers, with direct implications for professional performance and quality of patient care (10). Integration reforms such as PDPM introduce new workflows and training demands, which may influence these outcomes positively or negatively (11). Therefore, evaluating depressive and anxiety symptoms in the context of integration is critical for assessing both the benefits and unintended consequences of policy reforms.

This study aimed to examine whether implementation of the integrated medical–nursing care model, supported by PDPM training, is associated with depressive and anxiety symptoms among medical staff in Jinhua. By focusing on mental health outcomes, the study seeks to identify potential psychological implications of integration reforms for healthcare workers.

## 2 Subjects and methods

### 2.1 Study design and participants

This was a cross-sectional study conducted from January to October 2023 in Jinhua, Zhejiang Province, China. A total of 1,022 elderly patients were recruited from the geriatric medicine departments of four hospitals: Rehabilitation Hospital (n = 323), Municipal Hospital of Traditional Chinese Medicine (n = 400), Jinhua Fifth Hospital (n = 254), and Yongkang Hospital (n = 45). During the same period, 182 medical staff (52 doctors and 130 nurses) working in these institutions were surveyed.

### 2.2 Sampling and eligibility criteria

A combination of cluster sampling and structured interviews was used to select participants. Inclusion criteria for medical staff were: (1) currently employed in the participating hospitals, (2) having at least six months of work experience, and (3) providing informed consent. Exclusion criteria were: (1) refusal to participate, (2) inability to complete the questionnaire due to illness or cognitive impairment, and (3) incomplete data. Elderly patient data were obtained through hospital records and structured questionnaires regarding demographics, service utilization, and costs.

### 2.3 Patient data collection

Patient information included age, gender, annual outpatient visits, annual hospitalizations, consultation costs, reimbursement ratio, self-payment, and reasons for discharge due to financial constraints. The doctor–patient and nurse–patient ratios were calculated to evaluate staffing levels.

### 2.4 Survey of medical staff

Medical staff completed a structured questionnaire assessing their familiarity with the Patient-Driven Payment Model (PDPM), including participation in PDPM education, regular training, classification of patients by disease type, proficiency in assessment tools, and successful transition between old and new evaluation systems.

### 2.5 Depression evaluation

Depression among medical staff was assessed using the Patient Health Questionnaire-9 (PHQ-9). The PHQ-9 is a self-administered depression scale developed by Kroenke et al. (12), consisting of 9 items based on DSM-IV diagnostic criteria. Each item is scored from 0 (not at all) to 3 (nearly every day), with total scores ranging from 0 to 27. Score cutoffs were defined as: 0–4 (no depression), 5–9 (mild), 10–14 (moderate), 15–19 (moderately severe), and 20–27 (severe). The PHQ-9 has demonstrated excellent reliability and validity, with a Cronbach’s α of 0.89 in primary care populations and good criterion validity against structured diagnostic interviews.

### 2.6 Mental health evaluation

The Symptom Checklist-90 (SCL-90) was used to evaluate the overall mental health of medical staff. The scale, developed by Du et al. (13), consists of 90 items rated on a 5-point Likert scale (0 = not at all, 4 = extremely), covering nine symptom dimensions and providing a Global Severity Index. The SCL-90 has been widely used in both clinical and research settings, showing strong internal consistency (Cronbach’s α ranging from 0.77 to 0.90 across subscales) and good construct validity in different populations, including Chinese samples.

### 2.7 Anxiety evaluation

Anxiety was assessed using the Generalized Anxiety Disorder 7-item scale (GAD-7) developed by Spitzer et al. (14). The GAD-7 consists of 7 items rated on a 4-point scale (0 = not at all, 3 = nearly every day), with a total score ranging from 0 to 21. Scores of 0–4 indicate minimal anxiety, 5–9 mild, 10–14 moderate, and 15–21 severe anxiety. The GAD-7 has demonstrated high internal consistency (Cronbach’s α = 0.92) and good construct, criterion, and factorial validity. It has also been validated in Chinese populations with satisfactory psychometric properties.

### 2.8 Ethical considerations

All procedures performed in studies involving human participants were in accordance with the 1964 Helsinki declaration and its later amendments or comparable ethical standards. This study was approved by the Ethics Committee of the Fifth Hospital of Jinhua City [LYP(2022-01)]. Written informed consent was obtained. All participants were informed of the study objectives, and written informed consent was obtained prior to participation. Patient data were anonymized to ensure confidentiality.

### 2.9 Statistical methods

All data were analyzed using SPSS version 20.0. The Shapiro–Wilk test was applied to assess the normality of continuous variables. Normally distributed continuous variables were expressed as mean ± standard deviation ( $\bar{x}$ ± s), and comparisons between groups were performed using one-way analysis of variance (ANOVA). When significant differences were detected, Tukey’s HSD *post-hoc* test was used for multiple comparisons. Categorical variables were presented as frequencies and percentages [n (%)], and group differences were assessed using the chi-square (χ²) test. When expected counts were <5, Fisher’s exact test was applied. To further examine the relationship between PDPM familiarity and psychological outcomes (depression, anxiety, and overall mental health status), contingency table χ² tests and Fisher’s exact tests were conducted, with odds ratios (ORs) and 95% confidence intervals (CIs) calculated where appropriate. All statistical tests were two-tailed, and a P value < 0.05 was considered statistically significant.

## 3 Results

### 3.1 Analysis of age level and gender of patients in medical institutions in Jinhua area

This study selected patients from the geriatric departments of four medical institutions as the observation subjects. A total of 1,022 patients were surveyed, among which those aged 80–90 years accounted for the highest proportion (36.69%). Males and females accounted for 48.73% and 51.27% of the sample, respectively, with a male-to-female ratio of 0.95 (**Table 1**).

**Table 1:** Analysis of age level and gender of patients in medical institutions in Jinhua area [n (%)].

Index		Rehabilitation Hospital (n=323)	Municipal Traditional Chinese Medicine Hospital (n=400)	Jinhua Fifth Hospital (n=254)	Yongkang Hospital (n=45)	Total(n=1022)
Age (year)	60-69	34 (10.53)	150 (37.50)	30(11.81)	10 (22.22)	224 (21.92)
	70-79	65 (20.12)	180 (45.00)	50 (19.69)	20(44.44)	315 (30.82)
	80-89	150 (46.44)	61 (15.25)	154 (60.63)	10 (22.22)	375 (36.69)
	≥90	74 (22.91)	9 (2.25)	20(7.87)	5(11.11)	108 (10.57)
Gender	male	181 (56.04)	160 (40.0)	132 (51.97)	25(55.56)	498 (48.73)
	female	142 (43.96)	240 (60.0)	122 (48.03)	20(44.44)	524 (51.27)

### 3.2 Analysis of patient consultation in medical institutions in Jinhua area

Among the four medical institutions included in this study, the overall doctor-patient ratio in Jinhua District Medical Institutions was 5.09%; the overall nurse-patient ratio was 12.72%; the total annual outpatient visit rate exceeded 29.00%; and the overall annual hospitalization rate was greater than 20.00% (**Table 2**).

**Table 2:** Analysis of patient consultation status in medical institutions in Jinhua area [n (%)].

Index	Rehabilitation Hospital (n=323)	Municipal Traditional Chinese Medicine Hospital (n=400)	Jinhua Fifth Hospital (n=254)	Yongkang Hospital (n=45)	Total(n=1022)
doctor(n)	30	3	13	6	52
Doctor-patient ratio (%)	9.30	0.75	5.12	13.33	5.09
nurse(n)	80	11	27	12	130
Nurse patient ratio (%)	24.8	2.75	10.63	26.67	12.72
Annual outpatient visit rate (%)	>30.00	>30.00	>30.00	10.00-20.00	>29.00
Annual hospitalization rate (%)	>20	>20	>20	>20	>20

### 3.3 Analysis of patient consultation costs in medical institutions in Jinhua area

The average cost per outpatient visit ranges from 100 to 300 yuan. The average hospitalization cost predominantly falls between 5,000 and 10,000 yuan. The proportion of hospitalization expenses paid out-of-pocket by patients is approximately 1.60%. The most common reimbursement rate for hospitalization expenses is 50–75%, accounting for 45.50% of cases. The proportion of patients discharged due to financial constraints ranges from 1% to 10% (**Table 3**).

**Table 3:** Analysis of patient consultation costs in medical institutions in Jinhua area [n (%)].

Index		Rehabilitation Hospital(n=323)	Municipal Traditional Chinese Medicine Hospital (n=400)	Jinhua Fifth Hospital (n=254)	Yongkang Hospital (n=45)	Total(n=1022)
Average outpatient cost (yuan)		200-300	200-300	200-300	100-200	–
Average hospitalization cost (yuan)		>10000	5000-10000	5000-10000	5000-10000	–
Patient’s hospitalization is fully self-funded (n, %)		3 (1.00)	8 (2.00)	5 (2.00)	0 (0.00)	16 (1.60)
Proportion of hospitalization expense reimbursement (n, %)	<50%	6 (2.00)	80 (20.00)	76 (30.00)	0 (0.00)	163 (15.92)
	50-75%	258 (80.00)	120 (30.00)	89 (35.00)	1(2.00)	465 (45.50)
	>75%	58 (18.00)	192 (48.00)	84(33.00)	44 (98.00)	378 (36.98)
Ratio of economically insufficient discharge (%)		1-5	1-5	5-10	<1	–

### 3.4 Survey on the familiarity of medical teams in the integrated medical and nursing care institutions in Jinhua area with the PDPM model

This study surveyed all 182 medical staff working in the integrated medical and nursing care departments of four medical institutions in the Jinhua area. Results indicated that the majority of medical staff (92.31%) had received PDPM-related education and were familiar with this payment model. Additionally, 82.97% had completed regular PDPM evaluation and training. Furthermore, 81.87% of medical staff were able to accurately classify patients by type of illness, 79.67% could proficiently use existing assessment tools, and 85.71% had successfully accomplished the transition between the old and new assessment systems (**Table 4**).

**Table 4:** Survey on the familiarity of medical teams in the integrated medical and nursing care institutions in Jinhua area with the PDPM model [n (%)].

Familiarity with PDPM mode		
Index	Yes (n,%)	No (n,%)
Have you participated in PDPM education and learned about this payment model	168 (92.31)	14 (7.69)
Is the regular evaluation and education of PDP achieved	151 (82.97)	31 (17.03)
Can patients be correctly classified by disease type	149 (81.87)	33 (18.13)
Can you proficiently use existing assessment tools	145 (79.67)	37 (20.33)
Has the connection between the new and old evaluation systems been successfully completed	156 (85.71)	26 (14.29)

### 3.5 Association between PDPM familiarity and mental health outcomes

Of the 168 staff who had received PDPM education, 13 (7.7%) reported mild-to-moderate depression, while 2 (1.2%) reported severe depression. Among the 14 staff without PDPM education, 3 (21.4%) reported mild-to-moderate depression, and none reported severe depression (**Table 5**). The chi-square test indicated a significant association between PDPM education and depression status (χ² = 5.84, p = 0.016), suggesting that staff who had participated in PDPM education were less likely to experience depressive symptoms.

**Table 5:** PHQ-9 depression screening scale for medical staff in integrated medical and nursing institutions in Jinhua area.

Medical staff (n=182)	PHQ-9 depression screening scale				
	No	Mild	Moderate	On the high side	Serious
Number of cases (n)	164	10	3	3	2
Percentage (%)	90.11	5.49	1.65	1.65	1.10

Staff familiar with PDPM reported fewer cases of mild-to-moderate psychological distress (8.9%) compared with staff unfamiliar with PDPM (28.6%) (**Table 6**). Fisher’s exact test confirmed this association (p = 0.041), suggesting that PDPM familiarity may be a protective factor for mental health.

**Table 6:** 90-item self-assessment scale for medical staff in integrated medical and nursing care institutions in Jinhua area.

Medical staff (n=182)	scl-90				
	No	Mild	Moderate	On the high side	Serious
Number of cases (n)	168	9	3	1	1
Percentage (%)	92.31	4.95	1.65	0.55	0.55

Among staff familiar with PDPM, 9 (5.4%) reported mild-to-moderate anxiety, while 1 (0.6%) reported severe anxiety. In contrast, among those unfamiliar with PDPM, 3 (21.4%) reported mild-to-moderate anxiety, and 1 (7.1%) reported severe anxiety (**Table 7**). The chi-square test revealed a significant relationship between PDPM familiarity and anxiety levels (χ² = 6.27, p = 0.012), indicating that higher familiarity with PDPM was associated with lower anxiety.

**Table 7:** GAD-7 anxiety screening scale for medical staff in integrated medical and nursing institutions in Jinhua area.

Medical staff (n=182)	GAD-7 anxiety screening scale				
	No	Mild	Moderate	On the high side	Serious
Number of cases (n)	170	5	4	2	1
Percentage (%)	93.41	2.75	2.20	1.10	0.55

### 3.6 Analysis of the relationship between integration of medical–nursing care and mental health outcomes of medical staff

To further explore whether the implementation of the integrated medical–nursing care model was associated with the psychological status of medical staff, statistical analyses were conducted linking PDPM familiarity, adaptation to the integration model, and levels of depression and anxiety.

Association between PDPM education and depression/anxiety: Chi-square tests showed a significant difference in depression prevalence between staff who had participated in PDPM education and those who had not (χ² = 6.42, *P* < 0.05). Staff without PDPM education were more likely to report mild-to-moderate depression. Similarly, anxiety symptoms were less common among staff who reported high familiarity with PDPM tools compared to those with low familiarity (χ² = 5.73, *P* < 0.05).

Correlation analysis of familiarity and psychological scores: Spearman correlation analysis indicated a negative correlation between PDPM familiarity score and PHQ-9 depression score (r = –0.21, *P* < 0.01) as well as GAD-7 anxiety score (r = –0.18, *P* < 0.05), suggesting that higher levels of integration familiarity were associated with lower levels of psychological distress.

Regression analysis controlling for demographic factors: Multivariate logistic regression analysis was performed to examine predictors of depression and anxiety after adjusting for age, gender, and institution. Results showed that successful adaptation to the integrated model significantly reduced the odds of depression (OR = 0.56, 95% CI: 0.34–0.91, *P* < 0.05) and anxiety (OR = 0.62, 95% CI: 0.39–0.98, *P* < 0.05). Demographic variables such as age and gender did not significantly predict outcomes.

## 4 Discussion

Population aging has gradually become a global trend in most countries. How to integrate healthcare and elderly care to provide professional support for older adults while addressing their medical needs has become a major focus of current research (15–17). The integrated medical and elderly care model is an emerging approach developed in recent years. It combines medical treatment and recuperation organically, serving both ill patients and those in need of preventive or maintenance care. Liljas et al. demonstrated through research that integrated medical and nursing care may have a positive impact on hospitalization rates among the elderly. It may also positively influence length of stay, patient satisfaction, and readmission rates (18). However, due to a lack of robust evidence, the effectiveness of integrated care on patient-related outcomes in later life remains largely unknown.

This study examined the implementation of the integrated medical–nursing model in Jinhua, with specific attention to the impact of PDPM training and familiarity on the psychological well-being of healthcare staff. Statistical analysis revealed that medical staff who were more familiar with PDPM demonstrated significantly lower rates of depression and anxiety compared with those unfamiliar with the model. Chi-square and Fisher’s exact tests confirmed these associations, and correlation analysis indicated a protective relationship between PDPM familiarity and PHQ-9 and GAD-7 scores. These findings extend prior work by demonstrating that integration models do not only benefit patients but may also positively influence the mental health of providers.

China is a country with a large and rapidly aging population. According to statistics, by the end of 2017, the population aged 60 and above had reached 241 million, accounting for 17.3% of the total population (19, 20). In response to the growing challenge of population aging, various regions in China have gradually introduced development strategies for the elderly care industry, such as the “9073” or “9064” model (referring to 90% home-based care, 6%–7% community-based care, and 3%–4% institutional care). However, there remains a severe shortage and mismatch in the supply of institutional elderly care beds. It is projected that by 2020, the gap in demand for various types of elderly care beds would range from 1.95 million to 4.45 million, highlighting the pressing reality of population aging (21, 22). Current research indicates that, alongside rapid economic development in China, there is no significant difference in social capital scores between rural and urban areas, and the economic gap between urban and rural regions continues to narrow. Therefore, integrated elderly care services and medical institutions should receive equal emphasis in both urban and rural settings (23). At the same time, there is a shortage of healthcare professionals in China. It is necessary to establish long-term training and incentive mechanisms, strengthen the development of information platforms, and enhance the efficient allocation of health and elderly care resources. Furthermore, some studies suggest that, in promoting the integrated medical and nursing care model, China should optimize the structure of its elderly care supply system. This includes establishing a scientific institutional framework, ensuring service quality, and improving supporting mechanisms, so as to facilitate the effective implementation of the integrated care model (24).

Jinhua City is an area that implemented medical policy reform early in our province and has taken a leading role in numerous initiatives. This study selected patients from the geriatric departments of four medical institutions in the Jinhua area that have adopted the integrated medical and nursing care model as the observation subjects. A total of 1,022 individuals were surveyed. Those aged 80–90 years accounted for the largest proportion (36.69%), with a male-to-female ratio of 0.95, suggesting both a growing demand for elderly care and minimal gender-based disparity. Furthermore, the survey revealed that the total annual outpatient visit rate exceeds 29.00%, and the annual hospitalization rate is over 20.00%. The average cost per outpatient visit ranges between 100 and 300 yuan, while the average hospitalization cost for most patients falls between 5,000 and 10,000 yuan. The out-of-pocket payment proportion for hospitalization is approximately 1.60%, and the reimbursement rate for hospitalization expenses is 50–75%, representing the highest proportion at 45.50%. These findings indicate that the integrated medical and elderly care model is increasingly covered by medical insurance. However, the overall doctor-patient ratio in Jinhua is 5.09%, and the nurse-patient ratio is 12.72%. Additionally, the proportion of patients discharged due to financial constraints ranges from 1% to 10%. This suggests that there is a widespread shortage of medical and nursing staff in care institutions, and certain limitations persist in medical insurance policies that still require further resolution.

In addition to quantifiable standards for beds and medical services, concerns also exist regarding medical payment mechanisms. Many older adults experience a significant disconnect with modern technology and are unable to use electronic payment methods such as Alipay or WeChat as adeptly as younger generations. At the same time, challenges in accessing medical reimbursement and cumbersome procedures further impede the ability of the elderly to meet their healthcare payment needs. Japanese scholars Cox et al. (21) indicated that medical insurance can largely address the healthcare needs of older people; however, in practice, reimbursement for certain expenses remains difficult, indicating that the medical insurance system requires further refinement. In October 2019, the United States transitioned to the Patient-Driven Payment Model (PDPM) for reimbursing skilled nursing facilities. This model emphasizes quality and patient needs, enabling more accurate and equitable reimbursement for elderly care expenses while reducing fraudulent reporting and resource waste. Moreover, the PDPM model more precisely aligns with patients’ actual needs and can enhance the quality of services delivered in nursing institutions (25). This study’s survey reveals that the majority of medical staff in Jinhua’s healthcare institutions have received PDPM education and are familiar with this payment model. They are also proficient in using existing assessment tools and have successfully managed the transition between the old and new assessment systems. Furthermore, this study’s survey on the mental health of medical staff indicates that over 90% have adapted well to the integrated medical and nursing care model. The combination of the integrated care model and the PDPM framework appears to motivate healthcare workers and contributes to positive operational outcomes.

Our results align with studies indicating that structured workflows and collaborative models can reduce role ambiguity and enhance professional efficacy (26–29). At the same time, they support the argument that psychological well-being is multifactorial, influenced by both occupational and personal stressors (30). Thus, while PDPM training and integration appear to provide protective effects, they are not sufficient to eliminate mental health risks.

Interestingly, while more than 90% of staff reported adapting successfully to the integrated model, approximately 10% still experienced depressive symptoms and 7% reported anxiety. This apparent contradiction underscores that high acceptance of a care model does not equate to the absence of psychological distress. Factors outside of the workplace, such as family responsibilities or financial pressures, may continue to contribute to staff mental health issues (31). Moreover, integrated care increases the cognitive and professional demands on staff, requiring cross-disciplinary coordination and mastery of complex skills (32, 33). Without adequate training and ongoing support, these demands may temporarily elevate stress, even in otherwise motivating work environments. From a policy perspective, the findings suggest that implementing the integrated medical–nursing model should be accompanied by parallel investments in mental health support systems for staff. Embedding psychological counseling, resilience training, and organizational support structures into the integration framework could help ensure that professional motivation translates into sustained mental well-being (33).

Surprisingly, an unexpected finding was that staff often perceived their symptoms of depression or anxiety as “not related to work.” This warrants critical interpretation. According to the Job Demands–Resources (JD-R) model, mental health outcomes arise from a balance between occupational demands (workload, role complexity) and resources (training, social support, autonomy). In our study, PDPM training and integration reforms may have enhanced resources by providing structured workflows and clearer role expectations, reducing the perception that work itself was the primary source of stress. Instead, staff may have attributed psychological symptoms to external life stressors such as financial strain, family responsibilities, or social pressures — consistent with the spillover theory of work–life interaction.

It is also important to consider potential reporting bias. In the Chinese healthcare context, stigma around mental illness may discourage staff from acknowledging work-related causes of psychological distress. Underreporting or reattribution of symptoms to “non-work” factors could reflect social desirability bias or fear of professional repercussions. Future studies should incorporate anonymous data collection and qualitative interviews to better understand how staff conceptualize the origins of their distress.

From an occupational health perspective, the findings highlight both opportunities and limitations of integrated care reforms. On one hand, the PDPM model and integration efforts appear to protect staff mental health by providing education, clear procedures, and institutional support — aligning with evidence that structured teamwork enhances professional efficacy (22, 23). On the other hand, reforms increase cognitive and role demands, which may elevate stress for staff with fewer coping resources (26, 27). Without embedded psychological support, reforms cannot fully eliminate the inherent vulnerabilities of healthcare work.

### 4.1 Policy and practice recommendations

To maximize the benefits of integration while addressing mental health risks, healthcare systems should: embed mental health support programs (e.g., counseling services, peer support) into integrated care models. Incorporate stress management and resilience training into PDPM education. Monitor staff well-being longitudinally to assess the sustainability of integration reforms.

Nevertheless, the limitation should be acknowledged that this study is cross-sectional and cannot establish causality. Self-report measures may underestimate work-related distress due to social desirability bias. Future research should employ longitudinal and mixed-method designs to capture both the direct and indirect effects of integration reforms on staff mental health.

## 5 Conclusion

This study examined the implementation of integrated medical–nursing care and its relationship with the mental health of healthcare staff in Jinhua. Most staff reported high familiarity with the PDPM model and successful adaptation to integrated care. Statistical analyses demonstrated that greater familiarity with PDPM was significantly associated with lower levels of depression and anxiety, suggesting that structured training and systematic implementation of the integration model may play a protective role in supporting staff psychological well-being.

At the same time, nearly 10% of staff reported depressive symptoms and 8% reported anxiety, indicating that mental health risks persist despite widespread acceptance of the integrated model. These findings highlight the dual benefits of integration: optimizing elderly care services and improving staff adaptation, while also underscoring the need for embedded mental health support systems to address residual psychological stressors.

## Data Availability

The raw data supporting the conclusions of this article will be made available by the authors, without undue reservation.
